# Evaluation of mouse enteroids as a model for *Lawsonia intracellularis* infection

**DOI:** 10.1186/s13567-019-0672-9

**Published:** 2019-07-19

**Authors:** Talita Pilar Resende, Ramya Lekha Medida, Yue Guo, Fabio A. Vannucci, Milena Saqui-Salces, Connie Gebhart

**Affiliations:** 1 Department of Veterinary and Biomedical Sciences, College of Veterinary Medicine, University of Minnesota, St. Paul, MN 55108 USA; 2 Department of Animal Science, College of Food, Agricultural and Natural Resource Sciences, University of Minnesota, St. Paul, MN 55108 USA; 3 Veterinary Diagnostic Laboratory, College of Veterinary Medicine, University of Minnesota, St. Paul, MN 55108 USA

## Abstract

*Lawsonia intracellularis*, an obligate intracellular bacterium, is an important enteric pathogen in pig herds and horse farms worldwide. The hallmark feature of *L. intracellularis* infection is the proliferation of epithelial cells in intestinal crypts. A major limitation to the study of *L. intracellularis* infection is the lack of an in vitro model that reproduces the changes observed in proliferative enteropathy. Here we investigated the suitability of mouse enteroids as a model to study *L. intracellularis* infection. Mouse enteroids were microinjected with *L. intracellularis*, filter-sterilized *L. intracellularis* culture supernatant, or sterile cell culture media (DMEM). *L. intracellularis* antigen was detected in mouse enteroids by immunohistochemistry and was located mostly in the basal region of the epithelium. There was no differential growth of enteroids among treatment groups, and cellular proliferation was not increased in *L. intracellularis*-infected enteroids in relation to non-infected enteroids based on immunofluorescence staining. *L. intracellularis* infection did not induce changes in gene expression of *Ki*-*67* (proliferation marker), *Sox9* (marker for transit amplifying cells) and *Muc2* (marker for goblet cells). These results indicate that although *L. intracellularis* antigen is detectable in mouse enteroids, indicating susceptibility to infection, mouse enteroids fail to replicate the cellular proliferation and gene expression changes observed in proliferative enteropathy. Nevertheless, we have successfully demonstrated that mouse enteroids can be used to model days-long intracellular pathogen infection, serving as potential models for the study of other pathogens of interest in veterinary medicine.

## Introduction

Pig herds and horse farms worldwide are regularly challenged by proliferative enteropathy (PE). This disease is caused by *Lawsonia intracellularis* and is characterized by the thickening of the small intestinal mucosa due to proliferation of intestinal epithelial cells. PE causes diarrhea and compromised weight gain in pigs [1, 2]. In weaned foals, PE causes diarrhea and hypoproteinemia, occasionally resulting in death [3]. Other species are also affected by PE, including ratite birds, rabbits, non-human primates, rats and mice [4].

*Lawsonia intracellularis* is a Gram-negative bacterium that requires an intracellular culture system and a specific atmosphere to be propagated in vitro [5]. Traditional single cell cultures (cell lines) are permissive to *L. intracellularis* propagation, but they have failed to reproduce the increased cellular proliferation observed in PE-affected animals [6]. The lack of an in vitro model that represents the in vivo progression of PE has limited advancement in knowledge of the pathogenesis mechanisms utilized by *L. intracellularis*. Hence, identification of new alternatives for control and prevention of PE has been difficult, and pig producers and horse owners have been relying on available commercial vaccines (whole cell vaccines) and antimicrobials to prevent and control PE [7–10]. The costs associated with producing whole cell vaccines is reflected on the commercial price of the vaccines, hampering the access to some producers to the commercial vaccines, and, therefore preventing PE. In addition, the use of antimicrobials as growth promoters in swine production has raised concerns about the selection of antibiotic-resistant organisms in treated herds [11]. Therefore, a deeper understanding of PE pathogenesis is necessary for the development of novel non-antimicrobial based prevention and treatment methods such as recombinant vaccines or antimicrobial alternative strategies [11–14].

The currently held hypothesis is that *L. intracellularis* infects intestinal epithelial cells, especially in the crypt compartment, leading to cellular proliferation and decreased differentiation in goblet cells along with increased apoptotic events [4, 15, 16]. The mechanisms involved in epithelial changes during the course of PE are still unclear. Thus, knowledge about interactions between *L. intracellularis* and intestinal epithelial cells, determined using a controlled environment, could generate a better understanding of how *L. intracellularis* induces cellular proliferation.

A promising alternative to single cell cultures are tridimensional multi-cell type cultures, also known as organoids. One of the most relevant advantages of organoids in relation to the traditional in vitro single cell culture is the similarity of the organoids to their respective tissue of origin [17, 18]. Enteroids, small intestinal organoids, for instance, possess not only enterocytes, but also enteroendocrine cells, transit amplifying cells, goblet cells and stem cells. All these cells form structures with cell distributions that are similar to crypts and villi found in the small intestine, and possess a lumen where cell debris and secretion are shed continuously [17]. Enteroids have been recently used to study host–pathogen interactions of important enteric bacteria in human medicine [19–23], but they have not been investigated as a model for bacterial infections in the field of veterinary medicine [24, 25].

The objective of this study was to evaluate mouse enteroids as an in vitro model for *L. intracellularis* infection. Infected enteroids were monitored over time by size along with changes in expression of genes that have been reported to change during PE. We found evidence of infection with *L. intracellularis* in enteroids followed for up to 7 days post-infection (dpi). The genetic profile of infected mouse enteroids, however, diverged from the gene expression changes observed in PE.

## Materials and methods

### Growth and passaging of mouse enteroids

Mouse enteroid preparation and maintenance were performed as described elsewhere [26] (IACUC approval number 1606-33871A). The formulation of the enteroid culture medium was as described previously [27].

### *L. intracellularis* propagation

A *L. intracellularis* isolate (PHE/MN1-00, ATCC PTA-3457, Manassas, VA, USA) at low (≤ 20) passage [28] was propagated in McCoy mouse fibroblast-derived monolayers (ATCC^®^ CRL-1696™). McCoy cells were cultured as described elsewhere [29]. Cells were cultured in Dulbeco’s Modified Eagle Medium (DMEM, Gibco ThermoFisher, Waltahm, MA, USA) with 7% fetal bovine serum (FBS; heat inactivated, Corning™ 35011CV, Corning, NY, USA). McCoy cells at 30% confluency were infected with about 10^7^ *L. intracellularis* and incubated at 37 °C under a controlled microaerophilic atmosphere created with a mixture of gas containing 10% hydrogen, 10% carbon dioxide, and 80% nitrogen [30]. Seven days after infection, cell monolayers were mechanically lysed by passage through a 23-gauge needle coupled to a syringe after immersion in sterile 0.1% potassium chloride and then centrifuged at 200 × *g* to separate cell debris from *L. intracellularis* organisms. The pellets were discarded and the remaining supernatant was then filtered through 0.80 µm sterile filters (Millipore Sigma, Burlington, MA, USA) to remove any remaining McCoy cells and nuclei. After a final centrifugation at 8000 ×* g* at 4 °C the bacterial pellet was either used to re-infect McCoy cells, or to prepare the inoculum suspended in approximately 500 µL of its own supernatant and used as inoculum.

### Enteroid acclimation in microaerophilic and antibiotic-free conditions

As *L. intracellularis* growth requires special atmospheric conditions [30], we first tested whether the atmosphere conditions required for *L. intracellularis* growth would impact growth and development of mouse enteroids. One plate with enteroids was maintained under atmospheric conditions specific for *L. intracellularis* i.e., 10% hydrogen, 10% carbon dioxide, and 80% nitrogen [30] while another plate was maintained at 5% CO_2_ as a control. Both plates were incubated at 37 °C. Since *L. intracellularis* manipulation in vitro requires an antibiotic-free sterile system, antibiotics (Penicillin/Streptomycin) were excluded from both plates to assess sterility of the cultures. The plates were observed every 48 h for 3 weeks, with media replacement and enteroid passage as described previously.

### Pilot study—incubation and seed

**Figure 1:**
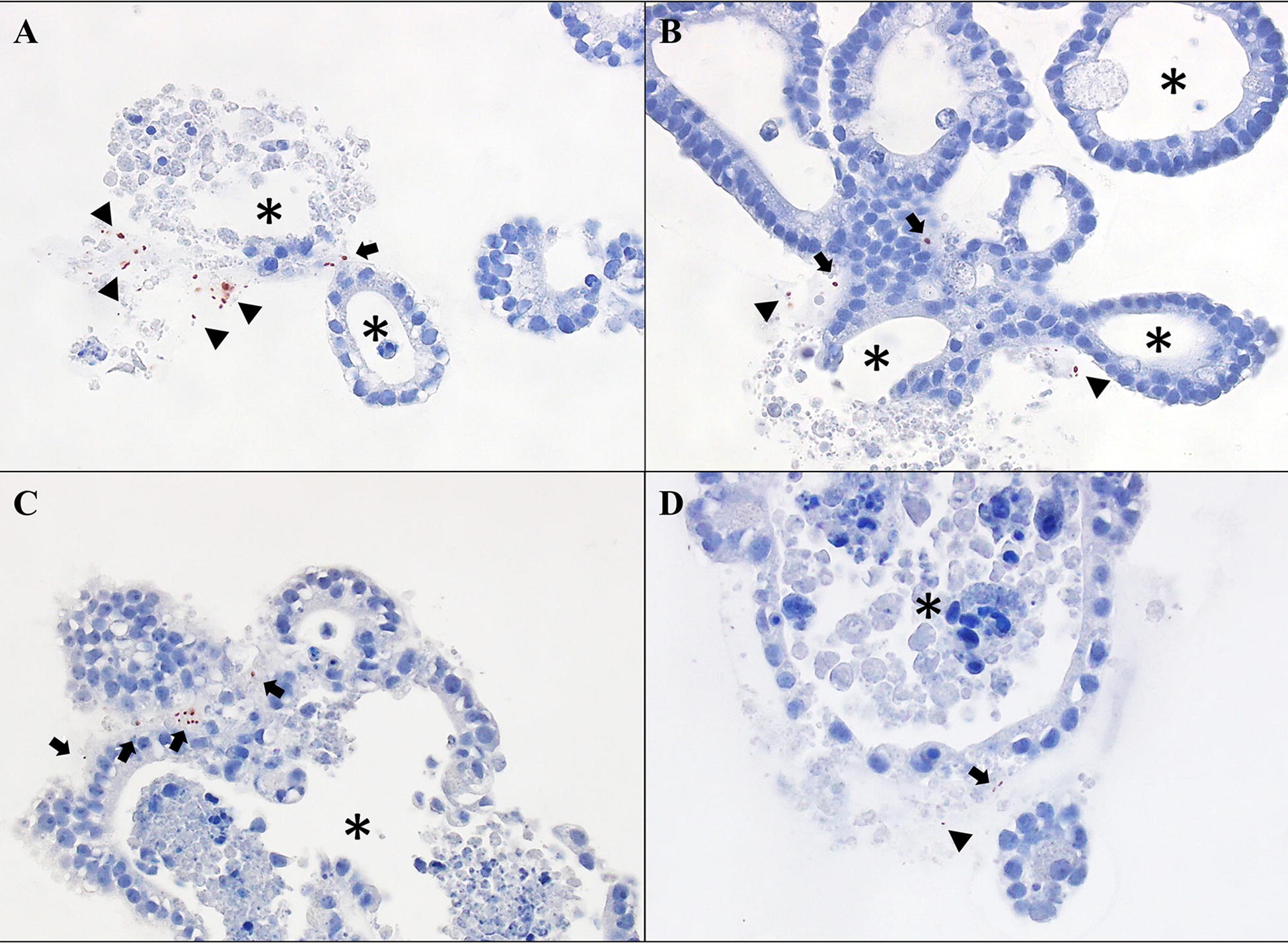
***L. intracellularis*** **infection in epithelial cells of mouse enteroids infected by the “incubation and seed” method. A** *L. intracellularis* antigen is associated mostly with cell debris (arrow heads), ×200. **B** *L. intracellularis* antigen is associated with  cell debris (arrow heads) and in close proximity to enteroid cells (black arrows), ×200. **C** *L. intracellularis* antigen is observed in close association with mouse enteroid cells (black arrows), ×200. **D** A few *L. intracellularis* antigens are observed in this image, some close to mouse enteroid cells (black arrow), and some associated with cell debris (arrow head), ×200. Enteroid lumen is indicated by an asterisk (*).

To standardize conditions for infection of mouse enteroids with *L. intracellularis*, we adopted the “incubation and seed” method [31]. Briefly, immediately after enteroid passage and before plating, enteroids were directly mixed with *L. intracellularis* suspended in 100 µL of enteroid culture medium (resulting in an inoculum with ~10^9^ *L. intracellularis* organisms/mL), with occasional gentle agitation, and incubated at 37 °C under special atmospheric conditions generated with a gas tank of 10% hydrogen, 10% carbon dioxide, and 80% nitrogen for 30 min. The infected enteroids were briefly centrifuged at 200 × *g* for 1 min, mixed with 50–100 µL ice-chilled Matrigel (Corning, USA), and then seeded into culture plates. Enteroids were harvested 15 days post-infection (dpi) by removing culture media and suspending the Matrigel in phosphate buffered saline (PBS). After centrifugation for 1 min at 200 × *g*, the pellet was suspended in 4% formaldehyde and incubated for 2 h at room temperature. Enteroids were then washed in PBS, centrifuged at 200 × *g* for 2 min, suspended in Histogel (ThermoFischer, USA) and placed into a biopsy mold. Histogel blocks were inserted into a cassette and maintained in 70% alcohol until they were processed for histological evaluation. Four-micrometer sections were placed onto glass slides and used for hematoxylin and eosin (H&E) staining and immunohistochemistry [32]. Although *L. intracellularis* antigen was detected by IHC in the infected enteroids by this method (Figure 1), we opted to employ the microinjection approach described below to facilitate contact of *L. intracellularis* with the cellular apical membrane in an effort to improve the level of infection.

### Enteroid infection with *L. intracellularis*

Mouse enteroids were cultured and passaged until a sufficient number of enteroids (average of 20 enteroids per treatment group, per time point) with 100 µm diameter were obtained for the trial. Wnt3a protein, which regulates the signaling pathways related to the cellular proliferation in the intestinal crypt compartment [33], was removed from the enteroid culture media at least 3 days before infection to enable cells to better differentiate. Twenty-four hours prior to infection, enteroids were divided into three treatment groups: DMEM: sterile culture media; SN: sterile-filtered *L. intracellularis*-culture supernatant; *L. intracellularis*: suspension with about 10^6^ *L. intracellularis* organisms/mL. Each enteroid received approximately 100 nL of each respective treatment. Treatments were placed directly into the lumen of the enteroids by microinjection using a microinjector (Nanoject II, Drumond Scientific, Broomall, PA, USA) operated by a trained technician. Experiments were performed in four independent replicates.

### Enteroid area monitoring

Microinjected enteroids were monitored for 1 week, from the day prior to infection until 7 dpi. Images were captured at 200× magnification using an inverted microscope coupled with a digital camera immediately after injection (0 dpi), and at 1, 3 and 7 dpi. The enteroid area was measured using an image software (NIH ImageJ software 1.46r, National Institute of Health, Bthesda, MD, USA). The relative enteroid area (enteroid growth) was measured by subtracting the enteroid area from the area recorded at its previous measurement.

### Enteroid harvesting and fixation

Enteroids from each treatment group were harvested at 1, 3 and 7 dpi and prepared for immunofluorescence. Matrigel was disrupted with a sterile pipet tip and the contents in each well were transferred to a 1.5 mL tube and centrifuged at 200 × *g* for 4 min. The supernatant was discarded, and the pellet suspended in 1 mL of PBS. This step was repeated two times to remove Matrigel. The resulting enteroid pellet was suspended and fixed in 4% paraformaldehyde for 1 h at room temperature. After fixation, enteroids were washed three times in PBS and centrifuged at 200 ×* g* for 4 min.

Fixed enteroids were embedded in Histogel (Richard-Allan Scientific HistoGel, ThermoFischer, USA) and then either placed in optimum cutting temperature (OCT, Tissue-Tek; Sakura Finetek, Beaver Creek, CO, USA) for preparing cryosections or dehydrated in 70% ethanol and embedded in paraffin.

### Immunohistochemistry

Paraffin-embedded sections (4 μm) on charged slides were used for IHC for detection of *L. intracellularis* using specific rabbit polyclonal antibodies and a previously described method [32]. Stained sections were evaluated by bright field microscopy.

### Immunofluorescence

OCT-embedded blocks were sectioned (4 µm), placed on charged glass slides and stored at −20 °C until immunofluorescence staining. Anti-Ki-67 immunofluorescence was performed as described elsewhere [6]. Briefly, slides were hydrated with Tris buffered saline (TBS) containing 0.1% triton-x (TBS-T) for 10 min and then blocked with 10% normal goat serum (ZC1213, Vector Laboratories, Burlingame, CA, USA) for 30 min to prevent non-specific binding. Sections were then incubated with anti-Ki-67 antibody (1:200 in TBS-T, CRM325B, Biocare Medical, Pacheco, CA, USA) for 2 h, followed by a 5 min wash in TBS-T and incubation with Cy3-labeled goat anti-rabbit antibody (1:250, ab97075, Abcam, Burlingame, CA, USA) for 30 min. All incubations were carried out at room temperature. Slides were washed and mounted with Prolongold (ThermoFisher, USA) and covered with glass coverslips. The number of Ki-67-positive cells and the total number of cells were counted in at least 5 random fields at 400× using an Olympus BX53 fluorescence microscope.

### Real-time quantitative PCR

Enteroids were harvested at 0, 1, 3 and 7 dpi, released from Matrigel as described above, lysed with 1.5 mL of TRIzol (ThermoFisher, USA) and stored at −80 °C until RNA extraction. RNA extraction was performed using chloroform and isopropanol precipitation following TRIzol protocols and then cleaning with the RNeasy mini kit (Qiagen, Germantown, MD, USA) according to the manufacturer’s instructions. Total RNA was quantified using a Nanodrop 2000 (Thermo Scientific, USA) and 400 ng of RNA were transcribed to cDNA using the High Capacity cDNA Reverse Transcription kit (Applied Biosystems, Foster City, CA, USA) with random hexamer primers.

Quantitative PCR reactions were performed using SYBR Green PCR Master Mix (Applied Biosystems) according to the manufacturer’s instructions. Amplification was performed with the following conditions: initial activation at 95 °C for 10 min, followed by 40 cycles of denaturation at 95 °C for 15 s and annealing at 60 °C for 60 s. Expression of the housekeeping gene *GAPDH* was monitored as reference. Relative gene expressions were normalized to *GAPDH* using the primer efficiencies. The expression levels of *Ki*-*67* (marker for cellular proliferation), *Sox9* (marker for transit amplifying cells) and *Muc2* (marker for goblet cells) in enteroids of all treatment groups were measured at 1, 3 and 7 dpi. Fold change in each case was calculated in relation to expression in enteroids from day 0 (control). All primers used are listed in Table 1.

**Table 1:** **Primers used for gene expression in mouse enteroids**

Target	Sequence of primers—forward	Sequence of primers—reverse
*GAPDH*	TCA AGA AGG TGG TGA AGC AGG	TAT TAT GGG GGT CTG GGA TGG
*Sox9*	CTG GAG GCT GCT GAA CGA GAG	CGG CGG ACC CTG AGA TTG C
*Muc2*	AGA ACG ATG CCT ACA CCA AG	CAT TGA AGT CCC CGC AGA G
*Ki*-*67*	TTT CAG GTC TCT GGA AGC AGT CA	ATC TCC ATA ATT GCT TTG ATT GCA

### Statistical analysis

Results are presented as a mean ± standard deviation of the mean (SEM) for each group. Two-way ANOVA followed by Geisser-Greenhouse correction was used to verify statistical differences with *p *<0.05 considered statistically significant. GraphPad Prism 8.1 software for Windows (GraphPad Software, La Jolla, CA, USA) was used to perform the analysis.

## Results

### Enteroid monitoring in microaerophilic conditions

There was no difference in growth or morphology between enteroids maintained for 1 week in the microaerophilic atmosphere relative to those maintained under regular conditions, i.e., 5% CO_2_ incubator (data not shown), indicating that growth conditions required for *L. intracellularis* do not impact enteroid viability or growth.

### Detection of *L. intracellularis* in mouse enteroids

**Figure 2:**
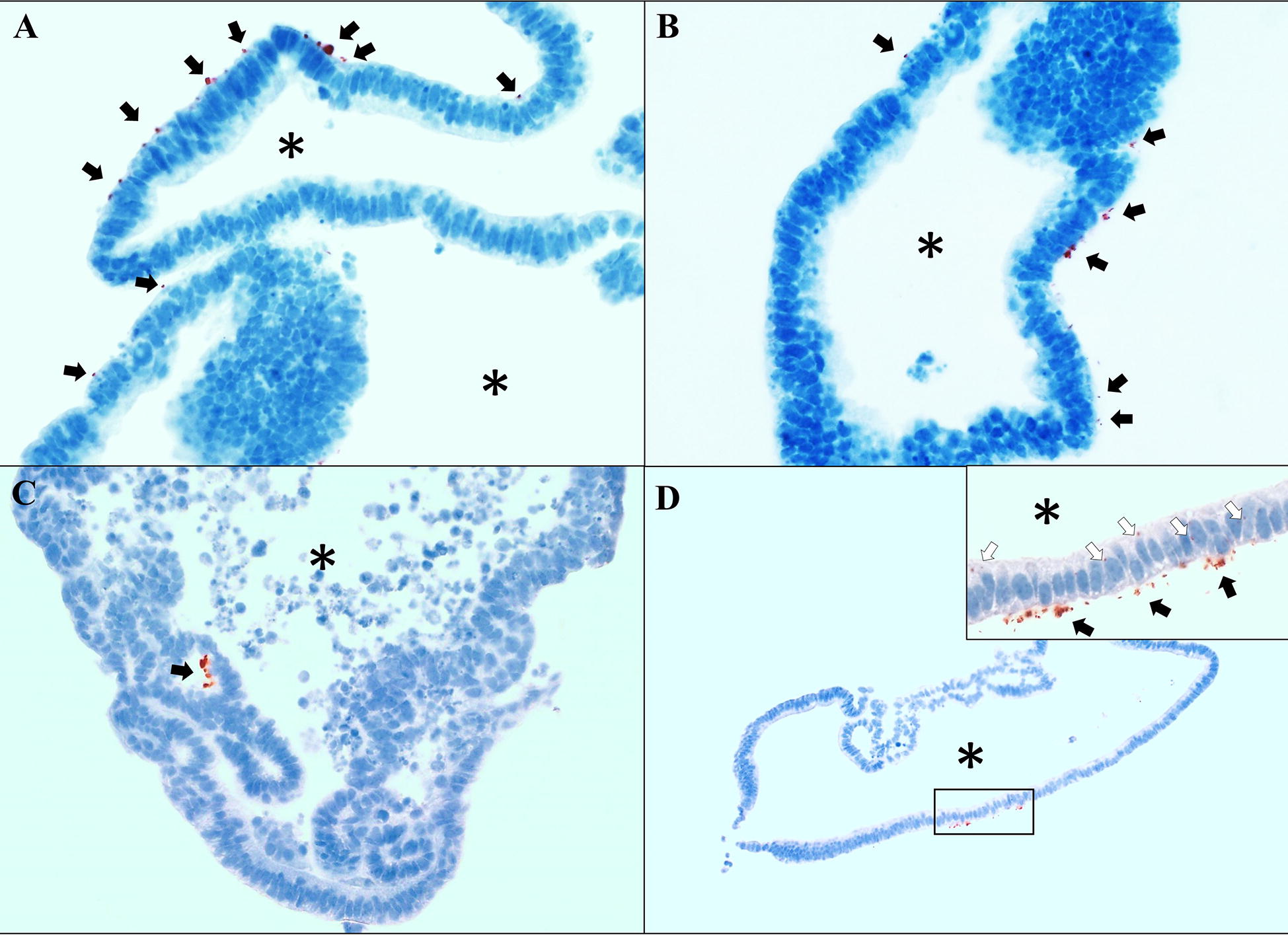
***L. intracellularis*** **infection in epithelial cells of mouse enteroids infected by the microinjection method. A**–**C** *L. intracelularis* antigen is observed on the basal region of mouse enteroids cells (black arrows). Most of the antigen is observed as amorphous clusters, instead of the typical rod-shaped morphology seen in vivo. ×400. **D** *L. intracellularis* antigen is observed on the basal region of mouse enteroids cells (black arrows) and in cellular cytoplasm (white arrows on the insert). Main image: ×100, insert: ×400. Enteroid lumens are indicated by asterisks (*).

Mouse enteroids were infected with *L. intracellularis* by microinjection. Enteroids were harvested and analyzed by IHC for *L. intracellularis* antigen at 1, 3, and 7 dpi. *L. intracellularis* antigen presence was observed at all time points, indicating successful infection with *L. intracellularis*. Unexpectedly, although *L. intracellularis* antigen was observed in the cytoplasm of enteroid cells, most of the antigen, especially at 7 dpi, was found in the basal region of epithelial cells (Figure 2). This is in contrast to the location of *L. intracellularis* in vivo, which is in the apical region of the epithelial cells [16, 32].

### Enteroid area was not affected by infection

**Figure 3:**
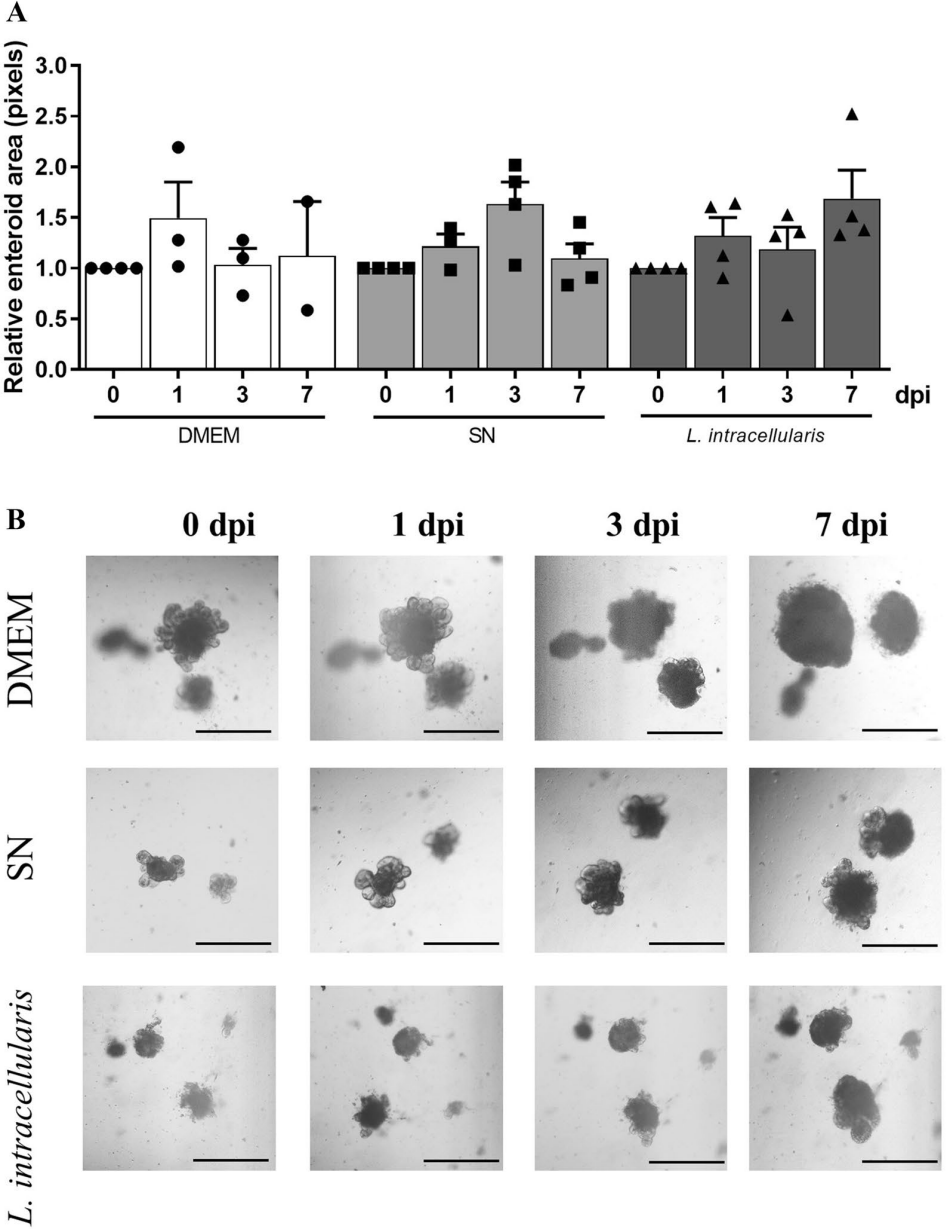
**Relative enteroid area over time. A** The relative enteroid area was calculated by the subtraction of the area of a given enteroid from the area of the same enteroid on the previous day. Bars represent mean ± SEM. **B** Bright-field images of each treatment group at 0, 1, 3 and 7 dpi. Scale bar 500 µm. Neg CTL, non-injected enteroids; DMEM, enteroids microinjected with sterile DMEM; SN, enteroids microinjected with filter sterilized *L. intracellularis* culture supernatant; *L. intacellularis*, enteroids microinjected with *L. intracellularis* in suspension.

To determine whether *L. intracellularis* infection would lead to increased size or secretory activity of infected enteroids, the area of enteroids in each treatment group was measured at 0, 1, 3 and 7 dpi. *L. intracellularis*-infected enteroids did not have higher area compared with SN or DMEM treatment groups (Figure 3A). Representative images of enteroids from each treatment group are shown in Figure 3B.

### Cellular differentiation

**Figure 4:**
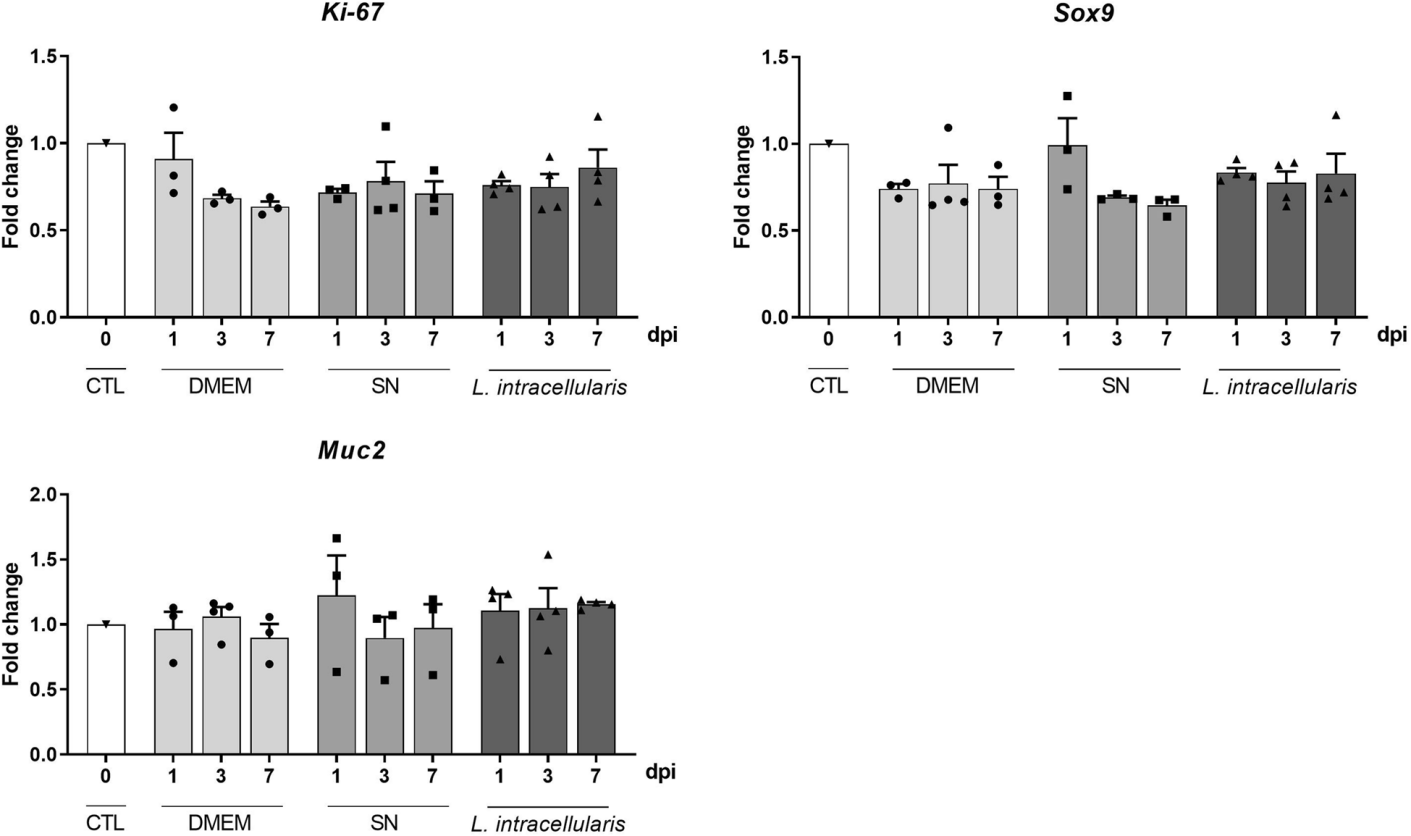
**RNA levels for** ***Ki*-*67***, ***Sox9*** **and *Muc2*** **as determined by RT-qPCR through a time course infection in mouse enteroids.** Fold change was calculated based on gene expression in enteroids at 0 dpi maintained under the same culture conditions but not subjected to microinjection (control). Bars represent mean ± SEM. Neg CTL, non-injected enteroids; DMEM, enteroids microinjected with sterile DMEM; SN, enteroids microinjected with filter sterilized *L. intracellularis* culture supernatant; *L. intacellularis*, enteroids microinjected with *L. intracellularis* in suspension.

**Figure 5:**
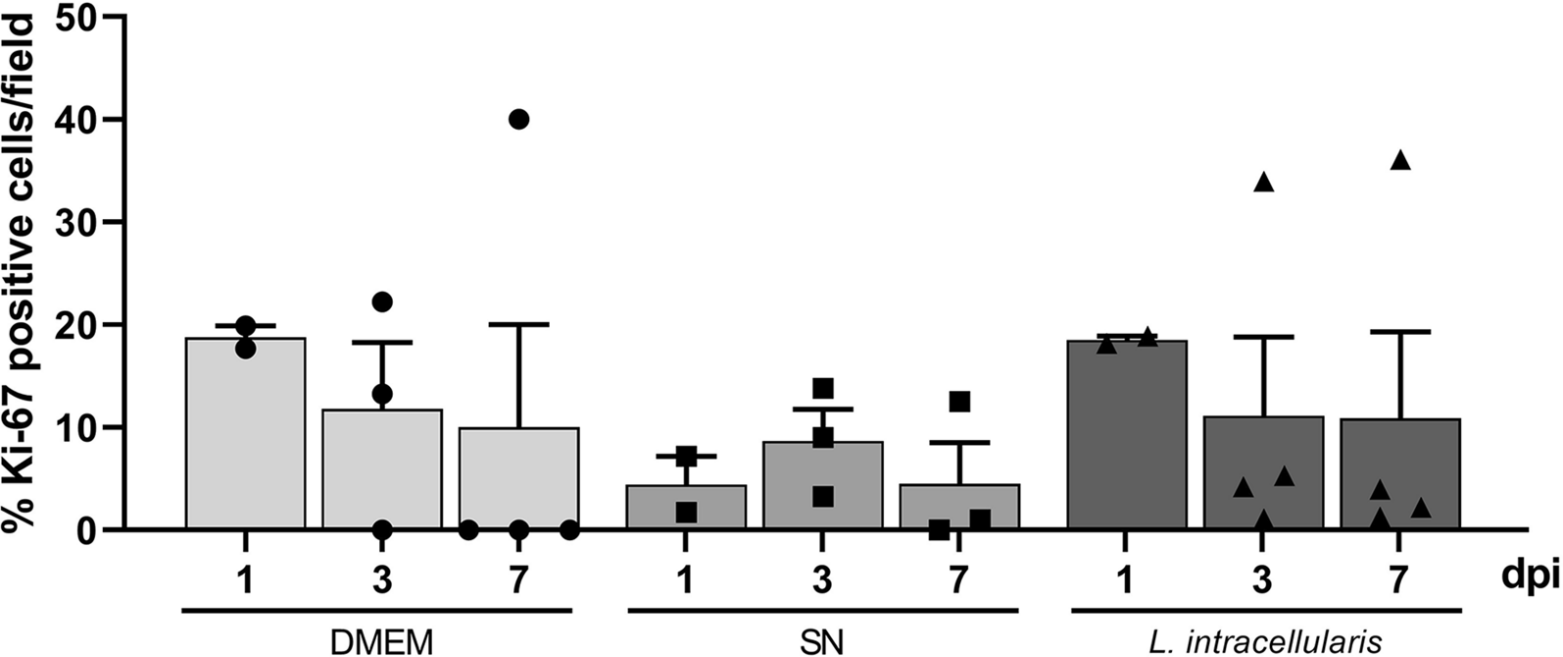
**Measurement of cellular proliferation in mouse enteroids.** Enteroid sections were stained for Ki-67 antigen by immunofluorescence and percentages of Ki-67 positive cells were calculated for all treatment groups. Bars represent mean ± standard error of the mean. DMEM, enteroids microinjected with sterile DMEM; SN, enteroids microinjected with filter sterilized *L. intracellularis* culture supernatant; *L. intacellularis*, enteroids microinjected with *L. intracellularis* in suspension.

To determine whether *L. intracellularis* infection induces changes in the proliferation and differentiation of enteroid epithelial cells, as observed in the swine intestine, we evaluated expression of *Ki*-*67*, *Sox9* and *Muc2* in enteroids harvested at 1, 3 and 7 dpi relative to expression in enteroids at 0 dpi. There were no significant differences in expressions of any of the genes analyzed in the treatment groups independent of the time point (Figure 4). Immunofluorescence staining confirmed that none of the treatment groups showed increased Ki-67 expression overtime, although in the *L. intracellularis* infected group considerable variability in the in Ki-67 expression throughout was noted (Figure 5).

## Discussion

Classical single cell culture systems have been extensively used in studies to understand host–pathogen interactions. Although they provide valuable information, these models are limited by their inability to represent the tissue organization observed in vivo since most of the single cell systems are either cancer-derived or transformed immortalized cell lines [19, 34]. These limitations make it difficult to assess the proliferative effects of *L. intracellularis* infection [6]. The mechanism by which *L. intracellularis* causes proliferation of intestinal epithelial cells remains unclear. Recently, the effects of *L. intracellularis* infection on the proliferation of monocultures of non-intestinal, non-epithelial cells, as well as intestinal epithelial cells were investigated under various culture conditions [6] and the findings supported previous observations that cell monocultures infected with *L. intracellularis* do not accurately represent proliferation observed in lesions in the intestine of affected animals. In vivo, *L. intracellularis* accesses the cell cytoplasm via the apical membrane and the organisms are observed in the cytoplasm throughout the course of PE. *L. intracellularis* is more frequently observed in crypt cells than cells of the villus [16, 35, 36]. Therefore, we hypothesized that enteroids would be a suitable model to further investigate *L. intracellularis* pathogenesis in vitro by providing polarized epithelial cell culture with all the crypt and villus epithelial cell types found in the mammalian intestine and similar cell exchange ratio to the in vivo intestine [37].

Enteroids were firstly developed in 2009 [38] and have been used to study intestinal morphophysiology as well as pathogenesis of some enteric diseases [21, 23, 39, 40]. Human enteroids can be obtained by culture of intestinal crypts isolated from biopsies. Mouse enteroids are more widely used in research as the ethical and biosafety regulations for human enteroids are more restrictive. Additionally, mouse intestinal physiology is well-characterized and molecular tools to study intestinal cell proliferation and differentiation are available. The presence of *L. intracellularis* DNA has been detected in feces of mice trapped in pig farms and in the surroundings of horse farms [41, 42], suggesting that mice are susceptible to infection. Furthermore, mice experimentally infected with *L. intracellularis* develop intestinal lesions that resemble lesions in affected pigs and horses, although the lesions in mice are less extensive, less severe and mostly localized in the large intestine when compared to the lesions in pigs and horses [42–44]. In addition, *L. intracellularis* has been successfully propagated in vitro using McCoy mouse fibroblast cells [6, 30, 45]. Based on this knowledge, we hypothesized that mouse enteroids may serve as a feasible model to investigate the progression of *L. intracellularis* infection and the mechanisms involved during cell proliferation.

Mouse enteroids in the tridimensional culture conditions, as spheres composed of polarized epithelium, have their cellular apical side oriented to the center of the enteroid. Hence, *L. intracellularis* infection in this study was performed by microinjecting the bacterial suspension directly into the lumen of the enteroid. Microinjection is a technique that has inherent limitations in controlling the bacterial inoculum, i.e., the exact number of bacteria each enteroid was exposed to. Although we plated enteroids to obtain an average of 10 enteroids per well, the actual number of enteroids per well and their size at the time of injection was found to be variable. These technical limitations may have contributed to the high variability observed in our results and constitute one of the limitations of using tridimensional enteroids as models for the study of luminal pathogens. Others have used mouse enteroids [20, 21] and human enteroids [22, 23] to study bacterial pathogenesis using microinjection. However, none of these studies evaluated infection times longer than 20 h [22, 23]. Because of the nature of *L. intracellularis* infection, longer durations, i.e., several days, are needed to observe the expected changes. To our knowledge, this is the first enteroid infection experiment reporting successful bacterial infection of enteroids for up to 7 dpi. Whether the length of the infection times had a significant effect on the number of bacteria present and variability on the evaluated parameters remains to be defined.

One of the most intriguing findings in the present experiment was the unexpected location of *L. intracellularis* in the infected enteroids. While *L. intracellularis* antigen was observed at all time points tested (1, 3 and 7 dpi), the antigen was mostly observed localized in the extracellular space proximal to the basal region of the epithelium of the mouse enteroids. In vivo, *L. intracellularis* organisms are consistently observed on the apical side of the cytoplasm at similar infection time points to those used in this study [16, 36]. A possible explanation for this difference in localization is that, in contrast to conditions in vivo, nutrients necessary for enteroid growth and maintenance are placed over the Matrigel, in a region that corresponds to the basal lamina in vivo and not directly in the enteroid lumen. We speculate that the gradient of nutrients on the exterior of the enteroid could be causing the *L. intracellularis* to move towards the nutrients to support their growth. It is also possible that conditions of the enteroid culture environment could result in less permissible conditions for *L. intracellularis* closer to the lumen. Another possibility is that the fibroblasts and immune cells in the basal region of the intestinal epithelium in vivo may have a regulatory effect on *L. intracellularis* traffic in the intestine, and the lack of those cells in the mouse enteroid culture would modify *L. intracellularis* traffic in vitro.

Immunohistochemistry for *L. intracellularis* is considered the gold standard method for diagnosis of proliferative enteropathy [32, 46]. In the present study, we used immunohistochemistry to verify whether the mouse enteroids, microinjected with *L. intracellularis* suspension (~10^6^ *L. intracellularis* organisms/mL), were infected and to visually demonstrate the level of infection in these microinjected enteroids. Our results were not only surprising in regards to the localization of *L. intracellularis* antigen, as discussed before, but also in regards to the amount and morphology of the stained antigen. Generally, *L. intracellularis* antigen stained by immunohistochemistry is observed as small bacilli in the cytoplasm of cell cultures [5, 47, 48] and in the apical cytoplasm of intestinal epithelial cells of naturally and experimentally-infected pigs. In the present study, instead of observing similar morphology of *L. intracellularis* antigen, we observed mostly the antigen as a “bundle” accumulated on the basal region of the enteroid cells. Nevertheless, occasional well-shaped *L. intracellularis* antigens were observed in the cytoplasm of some enteroid cells (Figure 2D), which is an indication that after the microinjection in the lumen of the enteroid, *L. intracellularis* organisms gained entrance into the cell cytoplasm. The events involving the changes in the morphology of the antigen and its final location in the basal side of enteroid cells remains unclear and may deserve more investigation. The homeostasis of the intestinal epithelium involves various signaling pathways of which canonical Wnt signaling is responsible for maintaining a balance between the pool of undifferentiated cells with proliferation capacity, named transit amplifying cells, and differentiated cells [33, 49]. In vitro, when enteroids are cultured in the presence of Wnt3a in the media, the transit amplifying cells stay undifferentiated for a longer period of time in relation to in vivo conditions [17], resulting in enteroids in a pro-proliferative state. In the present study, one of the main objectives was to verify the effects of *L. intracellularis* on cell proliferation, therefore we removed Wnt3a from the culture media 3 days before microinjection and during the experiment to allow the epithelial cells to differentiate, and better reproduce the proportion of proliferative cells of the normal intestinal epithelium. However, we failed to detect increased proliferation in *L. intracellularis*-infected enteroids by RT-qPCR or immunofluorescence. It is possible that the removal of Wnt3a 3 days before microinjection was not sufficient to reduce proliferation to a level where we could detect changes induced by *L. intracellularis*. Another possible explanation for this lack of difference in proliferation between treatment groups was the small amount of *L. intracellularis* delivered to the enteroid lumen, thus requiring a longer time for the bacteria to replicate in numbers to produce a detectable change in proliferation markers. The low amount of antigen detected in the microinjected enteroids indicate that *L. intracellularis* did not encounter an ideal environment for propagation as it does in McCoy cells and in the intestines of mice and other rodents [6, 30, 41, 43, 50]. The infection efficacy of enteroids derived from the intestines of more *L. intracellularis* susceptible species, such as pigs and horses, would help to confirm this hypothesis.

Changes in the area of enteroids can indicate an expansion on the number of cells or an increase in secretory activity. Secretory activity of enteroids has been demonstrated by swollen enteroids as a result of accumulation of luminal secretions after stimulation with forskolin [51]. In the present study, we expected to observe increased enteroid areas of infected enteroids that could be due to increased numbers of cells (proliferation) or secretory activity. In contrary to our expectations, we observed an increased volume from 1 dpi to 3 dpi on the SN group without changes in proliferation, suggesting the possibility of a secreted product with capacity to induce intestinal secretion. Further confirmation of this phenomenon is required.

*L. intracellularis* is a unique bacterium, requiring intracellular conditions and special atmospheric conditions for propagation in vitro. Little is known about *L. intracelllaris* survival in the cellular cytosol, or the host-cellular pathways that are disrupted during *L. intracellularis* infection. By using mouse enteroids as an in vitro model for *L. intracellularis* infectioin, our aim was to develop a tool to further define several aspects of *L. intracellularis* pathogenesis. The localization of *L. intracellularis* more abundantly in outside of the basal side of the enteroid epithelial cells may indicate nutrients or a microenvironment preferred by *L. intracellularis* and merits further investigation. Likewise, little is known about mouse enteroids maintained with a pathogenic enteric bacterium for a period longer than 24 h [20, 21]. More information is needed to determine if long-term infection of enteroids mirrors the dynamics of epithelium in vivo in terms of secretion, differentiation and apoptosis observed in chronic enteric diseases. Finally, yet importantly, although we were able to demonstrate infection of mouse enteroids, we did not observe the changes associated with *L. intracellularis* infection that have been noted in vivo in more susceptible species. Testing *L. intracellularis* infection in enteroids from other more susceptible species will provide further insight to species susceptibility to pathogenesis.

## Data Availability

The data collected in this project is shown in our results section. Further information can be available upon request.

## Abbreviations

cDNA: complementary desoxyribonucleic acid
DMEM: Dulbecco’s Modified Eagle Medium
FBS: fetal bovine serum
H&E: hematoxylin and eosin
IHC: immunohistochemistry
OCT: optimum cutting temperature
PBS: phosphate buffered saline
PE: proliferative enteropathy
RNA: ribonucleic acid
RT-qPCR: reverse transcriptase real time quantitative PCR
SN: filter sterilized *L. intracellularis* culture supernatant
TBS: triphosphate buffered saline
TBS-T: triphosphate buffered saline with triton X
